# Medical students’ experiences, perceptions, and management of second victim: an interview study

**DOI:** 10.1186/s12909-023-04763-7

**Published:** 2023-10-24

**Authors:** Tobias Browall Krogh, Anne Mielke-Christensen, Marlene Dyrløv Madsen, Doris Østergaard, Peter Dieckmann

**Affiliations:** 1 Copenhagen Academy for Medical Education and Simulation (CAMES), Herlev Hospital, Herlev, Denmark; 2 Department of Clinical Medicine, Copenhagen University, Copenhagen, Denmark; 3 Department of Quality and Health Technology, University Stavanger, Stavanger, Norway; 4 Department of Public Health, Copenhagen University, Copenhagen, Denmark

**Keywords:** Second victim, Second victim experiences, Second victim phenomenon, Second victim syndrome, Emotional distress, Adverse events, Work environment, Medical student, Medical education

## Abstract

**Background:**

The term *second victim* describes a healthcare professional who has been involved in an adverse event and feels wounded by the event. The effects of this experience differ. It can present as *second victim syndrome,* describing a wide range and degree of emotional and behavioural responses. Studies show that medical students can also experience second victim. The aim of this study was to elucidate medical students’ experiences, perceptions, and management of second victim and second victim syndrome and to describe possible learning needs around these issues.

**Methods:**

Thirteen medical students and two recent medical graduates participated in semi-structured focus group interviews. The interviews lasted 1.5–2 h and were audiotaped, transcribed, and analysed using Braun and Clarke’s six-step approach for thematic analysis.

**Results:**

Four main themes were identified: *contributing factors; current coping strategies; perception of own requirements and learning needs; wishes for the future healthcare system.* Students’ behavioural and emotional response to dilemmas were affected by stakeholders and practices embedded in the healthcare system. Students described patient-injury and unexpected events as triggers for second victim, but also harmful interactions with individuals and feelings of self-blame. Students’ coping centred around their network, formal offers, and separation of personal- and work-life. Students sought a clear definition of second victim and a desire for role-models. Students' wished to learn how to handle feeling like a burden to others, managing waiting time after patient complaints, and learning how to help second victims recover. Students emphasized the importance of the healthcare organisation understanding students’ needs and providing them relevant support.

**Conclusion:**

Students experience second victim as described in the literature. Students’ emotional responses were caused by classical second victim triggers, but also other triggers in the educational environment*: harmful interactions* and *self-blame*. Although some triggers differ from the second victim definition, these different triggers should be considered equally serious and acknowledged. We must aim to prepare students for future adverse events and emotional responses. The health organisation and healthcare professionals must support students’ mental well-being and contribute to ideal conditions for students' professional development and management of second victim as future physicians.

**Supplementary Information:**

The online version contains supplementary material available at 10.1186/s12909-023-04763-7.

## Background

The concept of second victim (SV) was introduced by Dr. Albert Wu in 2000 [1]. SV is defined as a healthcare professional (HCP) involved in an adverse patient event, medical error, or a patient-related injury who feels wounded by the event. The SVs suffering can present as a wide range and degree of emotional and behavioural responses affecting their professional and/or personal life [2–5]. How the terms *second victim* and *second victim syndrome* are utilized differs in the literature. Some definitions incorporate the involvement in an event, the response to the event and the impact of the response into the *second victim* term, whereas others specify the impact of the response (the suffering) as *second victim syndrome* [2–8]. Both terms will be applied in this study, with second victim referring to the involvement and response to an event and second victim syndrome referring to the impact of the response.

Several studies have sought to show the prevalence of SV. A systematic review showed a prevalence of SV varying from 10.4% to 43.4% among HCP’s [3]. A more recent study reported a prevalence of 62.5% in primary care and 72.5% in secondary care [9]. A German study reported that nine out of ten internal medicine residents didn’t know the term “*second victim*”. Nonetheless, 59% of the participants had experienced an SV incident [10]. A perception of greater personal responsibility for the event and poor patient outcome have been associated with a higher risk of developing second victim syndrome among residents [11, 12].

Few studies have explored medical students’ SV experiences. A recent Italian cross-sectional study found a prevalence of 4.6% [13]. Martinez and Lo described that medical students committing or observing others committing medical errors, may experience severe and persistent emotional distress similar to what HCP’s experience [14]. Medical students’ behavioural intentions regarding medical errors are shown to be influenced by three characteristics: students committing the error or observing others committing the error, the attendance of witnesses and the patient’s outcome [15]. Coping strategies among medical students consist of discussing adverse events with peers, other HCPs, or relatives not working in healthcare [16, 17].

The aim of this study was to elucidate medical students’ experiences, perceptions, and management of second victim/second victim syndrome and to describe possible learning needs around these issues.

## Method

### Study design and sample

The study was an explorative study based on semi-structured focus group interviews with medical students and recent medical graduates. This allowed participants to share sensitive issues and contributed to an in-depth understanding of our research question. In August 2021, 13 medical students and two medical graduates from the University of Copenhagen were recruited to participate in three semi-structured focus group interviews about SV.

Medical education in Denmark is prescribed to a total of six years. First three years as a bachelor’s degree programme and last three years as a master’s degree programme (Master of Science in Medicine). After graduation graduates enter a 1-year foundation training programme. For inclusion in this study, medical students had to be enrolled in the Master of Science in Medicine and have completed the mandatory five-week clinical rotation in the first year of the Master programme. Recent graduates were eligible if they hadn’t started their 1-year of foundation training. The recruitment took place through social media and oral requests of people in the authors’ networks. Participation was voluntary, and students were assured of full anonymity. Before the interview all students received information regarding the project and a short introduction to the SV phenomena and syndrome. They then signed a declaration of consent. The project was submitted to the Danish Committee of Health Research Ethics for ethical approval*.* The study was exempted from ethical approval since no patients were involved (Journal no.: 21047263).

Each focus group comprised five students. All interviews were conducted by two authors (TBK, AMC) and lasted 1.5–2 h. All students received a demographic questionnaire prior to the start of the interview. The interview guide (Appendix 1) included questions designed to elucidate the students’ knowledge, experiences, responses, and coping strategies about the SV phenomenon and syndrome. Furthermore, suggestions of potential learning needs and teaching methods about SV were discussed. The interviews were audiotaped and transcribed by a research assistant with experience in transcribing and TBK. Prior to analysis, transcriptions were compared to audiotapes for accuracy. Selected citations were translated word-to-word by TBK from Danish to English focusing on understanding and meaning. The research team reviewed the final English citations to verify that the understanding and meaning were kept compared to the Danish citations.

### Analysis

Braun and Clarke’s six-strep approach were used for thematic analysis [18]. The transcripts underwent a thorough readthrough to generate a familiarization with the material. One author (TBK) read all three interviews and two authors (AMC, PD) read one interview each. Next, the authors independently generated initial codes based on the material during a second read-through. The codes were generated by summarizing key-points from the transcripts. All codes were compared and discussed between the authors until consensus. The codes were organized and formed into four themes with subthemes. Themes were reviewed between all authors in relation to codes and finally clear definitions and names of the themes were generated.

## RESULTS

Demographic characteristics showed that students’ age ranged from 24 to 28 years old. A total of 15 students participated in the study, 11 students were females and four were males. All students had completed the first year of the Master programme. 13 students were in their 4th to 6th year of the medical education, and two were recent graduates. The students were a homogenous group in terms of having completed long clinical rotations. 14 students had a student-relevant healthcare-related job e.g., phlebotomist or clinical assistant. Students used their experiences from their student-relevant jobs in the interviews. Seven students indicated that they knew the term SV before participating in the interview. The SV experiences described by students were predominantly episodes concerning patients *dying*, *suffering severe illness* or being *violent* against the healthcare provider. Less common events were *error-making* or *poor performance* in the presence of a HCP.

**Fig. 1:**
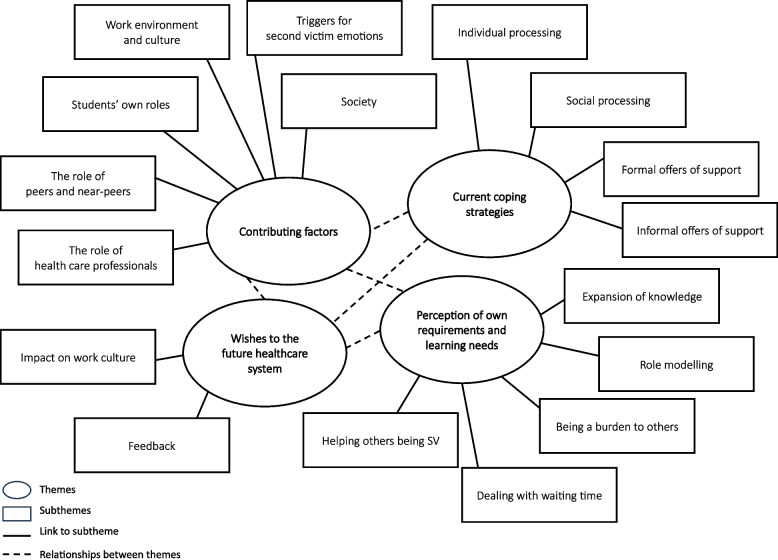
Mind-map themes and subthemes

Four main themes with subthemes were identified from the analysis: *contributing factors; current coping strategies; perception on own requirements and learning needs; wishes for the future healthcare system.* Themes and subthemes are shown in Fig. 1. Each theme and subthemes are examined. Some themes and subthemes are supported by selected relevant citations. Additional citations to themes and subthemes can be found in Appendix 2.

### Contributing factors

Students described different contributing factors in relation to their experiences. These contributing factors included the role of others, the physical environment, psychological, and social factors.

#### The role of health care professionals

Students’ experiences and perceptions of HCP’s attitudes and actions had an impact on how students navigated in a context, how they performed, and how they experienced their self-efficacy:

*“[…] the physician asks me […] if I want to place the cannula, and I say ‘yes, but you have to watch over my shoulder’, and she gets annoyed and says ‘no, I don’t want to, I don’t have time for that, I’ll take charge’. And I just thought yes now I’ll never do this again […] I’m with her again another day, and she asks me ‘have you learned to place a cannula yet?’ […] I choose to say yes, because I was so tired of being caught unaware all the time, […] So, I said yes, and totally messed it up of course, and she gets mad at me […] it was really one of these situations that affected me, because I was like ‘I simply can’t do anything right during the clinical rotation’. I tried to be the humble one and ask for help, and I really wanted to, but I also must learn, so I must try, and then I tried being the overly confident one, and messed it up again […].” (Interview 3).*

Physicians, noteworthy senior physicians, were portrayed predominantly in a negative way and almost as villains by students when it came to bringing up adverse events or other uncomfortable situations. The portrayed physicians provided little support or guidance and students' willingness to request help when needed reduced. The negative portrayal affected students’ behaviour when they heard about or experienced questionable actions by physicians, making it difficult for them to try to re-establish a positive interaction in the future. Furthermore, students described a correlation between physicians’ level of experience and their ability to be empathic. Years of experience and resilience affected the physician’s ability to understand how adverse events could affect inexperienced students in the current healthcare system. The discrepancy impaired students’ desire to discuss experiences and emotional distress with physicians. Nonetheless, physicians were also portrayed positively in situations where they created epiphanies for students. This could be by sharing advice or experiences, and thereby making it easier for the students to navigate in future contexts:

“*[…] there was an anaesthesiologist, he told us, […] x numbers of residents will suffer adverse health effects due to stress or so, leave work crying in the three initial months of their residency […]. He made a virtue of telling us about these things, and then he said ‘when you are graduating, then go to work and just do your best, and don’t be afraid to ask for help. And if you do so, then you shouldn’t walk around blaming yourselves too hard about not always winning’ […] you will always make errors, and it has stuck in my mind […], I’ll do my best every time and accept that my competencies only reach to here and now you need to receive help.” (Interview 2).*

#### The role of peers and near-peers

Peers or near-peers influenced the level of sharing and coping. The students’ equal relationship made it easier to discuss difficult academic topics, clinical situations, and procedures. Students felt a greater acceptability and understanding from peers than physicians. However, a competitive environment among students was described to affect the request for help negatively. Students described how being judged by peers when asking for help led to greater suffering.

#### Students’ own roles

The students’ appearances in others’ eyes had an influence on how they acted in different contexts. They were particularly self-conscious about their professional skill set, appearance, and reputation among others. Students feared sharing vulnerable or difficult experiences and emotions as well as asking for help in difficult situations. Students felt being vulnerable could affect their possibility of clinical learning by being less suitable for assignments which other students would then obtain.

#### Work environment and culture

Work environments and cultures at the hospitals and the university had a strong impact on students’ tendency to process adverse events and emotional distress.

Work environments characterized by high workload and lack of resources was mentioned as negative influences. Seeing junior physicians in their first year of foundation training with heavy and stressful workloads and no-one caring except the other new colleagues, made a huge impact. Students described how changing departments during clerkships provoked emotions of lacking a base, which made it difficult for students to create an affiliation, build relationships and seek help among peers and HCPs. Lack of acknowledgement and feedback from HCPs during clerkships were perceived negatively by students. In addition, the departments seemed to focus on organisational goals and less on valuable learning opportunities for the students. This made students less likely to engage in their own learnings experiences and impacted their tendency to talk about emotions and adverse events.

The students’ perceptions of the existing error culture in the hospitals affected them. They were concerned about making errors and speaking up, because of the unknown consequences, especially when human lives were involved. Nonetheless, students were aware of errors being inevitable in the job as a physician. Strong emotions of insecurity hit the students already before making their first error:

*“[…] I’m thinking ‘can I handle it?’, like am I mentally stable to handle it? It affects me a lot, and it’s not okay, […] the ideal scenario is a culture, where you can say, that it’s okay to make errors, people make errors and we catch each other, like the system should catch you […]. You have been told, you can be a physician from the university, then you need to feel the system catches you in the other end or supports you. It can’t be each individual responsibility.” (Interview 2).*

The culture at the university influenced students’ perceptions of error-making and adverse events. A culture of perfectionism created presumptions about the real world and could incite feelings of insufficiency among students.

#### Society

The students perceived society as having a demand for nearly flawless physicians who aren’t allowed to make errors, especially because real patients would suffer. This made students feel pressured and increased their fear of making errors in the future. Stories of physicians being publicly shamed by media outlets were mentioned as a worst-case scenario. Thoughts on receiving a patient complaint and waiting while it is processed were a burden to the students.

*“[…] the expectations towards us are already high, and if you hear about physicians, who have made some sort of error and it hits the media, and then they are just treated mean right? Like, I think it’s through that we get the ‘I’m not allowed to make errors’, because it can affect people.” (Interview 2).*

#### Triggers for second victim emotions

Three types of situations were described as triggering SV emotions among the students. First, experiences where someone conducted an action resulting in patient injury, or when an unexpected patient-related condition occurred. One student described this with the example of witnessing a young mentally handicapped woman going into cardiac arrest and unexpectedly dying during resuscitation. Second, experiences where patients acted violently against the student, which incited the feeling of self-blame. One student described being punched by a patient hallucinating. When students described these experiences, they tended to blame themselves for not fulfilling their work assignment or not taking good enough care of the patient. Third, experiences where interactions with other HCPs or peers caused an uncomfortable experience triggering emotional distress. One student experienced this when reporting to a nurse about the lack of sleep in a patient experiencing delirium, and the nurse disrespectfully ignoring the student’s observation and request for help.

The severity of the experience had an impact on students’ likelihood of discussing it. Students tended to devalue minor events even though their related emotions were quite strong. This was due to a fear of being judged as vulnerable or being seen as a burden by others. Students also questioned the legitimacy of their emotions. They remarked how others dealt with SV experiences “perfectly”, without any need for further help or need to verbalize their experiences. Students expected themselves to handle their SV experiences just as perfectly. Furthermore, when some students had an emotional response to an SV incident, it incited guilt for taking away the focus from patients. Students illustrated this by seeing themselves as “just being at work” while patients were the ones experiencing terrible, life-threatening, and traumatic events.

### Current coping strategies

When students were asked about coping with adverse events and SV experiences, their coping strategies were mainly centred around two aspects: individual and social processing, either using more formal offers of support or their informal network.

Students saw the relevance of taking care of themselves during clerkship. One student described this with a concept like compartmentalization where work- and private-life were consciously separated from each other to minimize the amount of work-related distress spilling over to other areas of life. Another student illustrated how to protect oneself prophylactically before the adverse event had even occurred:

“*[…] when I enter the hospital, I imagine that together with my uniform, I put on a teflon suit, in such way that it [experiences] don’t enter the inside of me […]. Of course, I take it in, but not on a personal level, where it’s about who I am, but more like ‘what could I do different’, trying to resonate things.” (Interview 3).*

Students described experiences where formal support was offered from the workplace. Participation in these offers was either voluntary or mandatory and would be held with more experienced colleagues, or psychologists. Voluntary offers had lower participation because students often questioned the need for support, once they were past the immediate experience. Mandatory offers of support were appreciated, because students saw the value of defusing and follow-up on the incident prospectively. Furthermore, students with a resourceful network tended to use this for coping. Students used different parts of their network for either clinical or emotional aspects of the issue:

*“[…] I do it when it’s clinically challenging with my fellow students and colleagues, and when it’s emotionally challenging with my good [non-medical] friends, […] it does something else, and it doesn’t have a medical focus with ‘oh, the blood pressure was this and that, and what did you think about then?’, but much more ‘how did you feel and why?’.” (Interview 2).*

### Perception on own requirements and learning needs

Students’ assessment of learning needs concerning SV centred around two main features: expansion of knowledge and role modelling, and three minor features; learning how to handle the feeling of being a burden to others; finding a way of dealing with waiting time in cases of patient complaints; learning how to support others dealing with being a SV or second victim syndrome.

The term “*second victim”* was not an active part of the students’ daily language, even though they could relate to the concept. When students were asked about their own learning needs, they pointed out the importance of a clear definition of SV and learning more about the concept. Students suggested this would create more openness and would make it easier to relate to being a SV. In addition, it could strengthen a culture, where vulnerability and sharing emotions would be allowed. In addition, students requested more education on consequences of making an error and the process of handling errors.

Students had a desire for role models. Being able to have role-models sharing how they experienced, felt, and handled being a SV and to give advice, would help students legitimize their own experiences and emotions. The role-model’s ability to be relatable were important and were related to the HCPs level of experience. Some students found experiences from junior physicians more relatable, while others saw greater value in knowing that SV and second victim syndrome could also affect experienced HCPs:

*”I think it’s much more valuable to hear from a junior physician and not an old toad [senior physician]. But a junior physician, because that’s what you see, that’s me in a moment […] whether it’s on video [learning material] is a brilliant idea, also where you can focus on ‘I had these problems, I handled them like that, and this is what it led to’.” (Interview 2).*

Students were occasionally declined when asking for help or supervision. In these situations, students tended to feel like a burden to HCPs, which made them stop seeking help and try to compensate for their perceived failure by increasing their work ethos in future tasks. This created a foundation for a learning need on how to handle being a burden. Students additionally requested more education on receiving a patient complaint, and what would happen in the aftermath of the complaint. This also created a learning opportunity on how to deal with the possible emotional distress linked to the waiting time before the complaint’s verdict:

*“I think generally that there’s not enough education on, what happens when you make an error, or if someone believe you made an error, which is the most common right? That you receive a [patient’s] complaint because someone believe you made an error and maybe you didn’t, and then 100 years passes, and you don’t hear anything [during the process] […]. I think it would be nice if you got to know more about how acceptable it is […].” (Interview 3).*

Lastly, students acknowledged that they also had a responsibility of offering support for other SVs. Students verbalized the need for communication skills training on how to talk about SV including how to actively support others recovering from an incident or syndrome.

### Wishes for the future healthcare system

Students emphasized the importance of maintaining a focus on SV by enhancing the educational focus on the topic and ensuring recurring teaching of SV throughout medical education, both at the university and in clinical training, including introducing and expanding SV to HCPs in general. Students especially pointed out the importance of implementing the SV definition and its meaning in the healthcare work environment, to make it clinically relevant and not just an academic definition. The HCPs and educational supervisors were seen to play an essential role in students’ educational environment and future shaping of the work culture. Emphasizing the seriousness and importance of knowledge about SV and second victim syndrome for future generations by educating with real-life and well-known events were also suggested.

*“You imitate what you see out there, […] because you look up to the physicians. […] you turn into them at some level, and that’s why it’s so strong, if it’s a senior physician who’s going through his/her own errors [openly in front of students]. […] we are so hierarchical, and if the culture is settled there [at the top], then it’ll migrate down. I think, it’s more difficult to change something from the bottom, because we don’t have any power, we can say all kinds of things, but we don’t have as much power as at the top.” (Interview 1).*

Furthermore, students wished to receive more feedback on their work and work ethic from HCPs to prepare them for difficult situations in the future. Students felt medical education had too much focus on finding the correct answer and less about the process leading to the answer.

## Discussion

Our results show how different contributing factors and triggers affect students’ responses to and assessment of adverse events and SV incidents, as well as how students currently cope in the aftermath. The role of HCPs, peers, and the students themselves in incidents has a high impact on the outcome. In addition, students experienced that work environments and their perception of society’s expectations towards HCPs affected their actions. Students had a clear idea of some potential learning needs specifically related to changing the system, including focusing on role-modelling and integrating SV in clinical practice. However, they lacked vision on handling individual struggles related to being a burden, patient complaints and supporting other SVs.

A primary finding was students’ relationship to the concept of SV, and how this played an important part of legitimizing their experiences and emotions. Specifically, legitimizing when they are affected by an experience, when they feel like an SV and when they have the right to use the label. Our findings are in line with previous studies [13, 14, 19].

In our study students described three different types of events that triggered SV emotions: patient injury or unexpected negative events, feelings of self-blame, and harmful interactions. Patient injury and unexpected negative events are in line with Wu’s description of an SV [1], while self-blame and harmful interactions are not covered in this definition. The emotional distress students describe regardless of a SV-defined event or not seems comparable to the emotional distress that HCPs describe in the SV literature [2, 3, 8, 9, 20]. The literature suggests that the ethical processing and social interactions students develop in response to dilemmas during their education, shape their reactions in their future professional career [21]. Therefore, the events and the emotional distress students encounter might not always be classified as SV, but they are still likely to influence students’ adjustment into the healthcare system, as well as their future conduct and emotional response with SV experiences as physicians.

Students seek a clear definition of SV. Dilemmas concerning the legitimacy of students’ emotions arise when the emotional aspects aren’t valued equally with the professional aspect. None of the different definitions define the SV according to severity or exact specificity of the experience. This allows individuals to self-reflect and interpret their experience and emotional response. The reason why students might seek a clear definition may be to create a frame of reference to justify the verbalization of their experiences and emotions. This would allow them to define themselves as a “true” SV to process and cope with their situation.

It is valuable to teach students to use themselves as the frame of reference instead of an academic definition. It is important that they learn how to deal with being a SV or experiencing second victim syndrome in a way where patient harm is avoided, and students’ well-being is restored. This may also contribute to legitimizing HCPs emotions and vulnerability in the clinical practice generally. Medical educators and HCPs must be made familiar with students’ experiences and perceptions of SV phenomenon and syndrome to implement the emotional aspect into the work environment, clerkship, and medical education.

A second finding from our study was how significant stakeholders and practices embedded in the healthcare system contribute to students’ behavioural and emotional response to dilemmas. Martinez and Lo described how role models’ responses to errors might have an important impact on students [14]. The literature shows similar findings of residents and attendings influencing students’ development of professional identity [17]. Furthermore, poor role-modelling has been reported to impact students’ well-being negatively [22]. Students’ self-reported awareness and confidence regarding adverse events and errors increased after a session containing video vignettes of physicians sharing experiences with errors [23].

In our study students described how strong clinical hierarchies and interactions with HCPs impacted them. Harmful interactions caused negative emotions such as frustration, discomfort, insufficiency, and loss of self-efficacy. The students described how these emotions made them feel like a burden to HCPs, which affected their later actions. Tendencies to stay passive or to overcompensate with activities were described. Students felt vulnerable, when placed in new settings with new colleagues and procedures. Many new aspects need to be dealt with when students have sometimes-limited clinical knowledge and knowledge of work procedures and the environment. In addition, when students experienced the shortage surpassed the reserve of energy among the HCPs, they felt neglected, denied help, or put in difficult clinical situations without sufficient supervision. Clinical environments with harmful interactions may create situations, where students choose not to ask questions, reflect openly, speak up, or ask for help when needed [14, 16]. This can be quite harmful concerning the importance of the early stages of students’ formation of professional habits and identity. A previous study has found difficult encounters with other HCP as one of the three most stressful events for students and that difficult clinical events affected their well-being [16]. A cross-sectional study showed that only 13.4% students thought the culture during their rotation made it easy to disclose medical errors [24]. Furthermore, another study identified that 56% of students didn’t speak up about patient safety issues during a critical situation, and students who did not speak up previously easily developed a habit for staying silent [25].

The development of a professional identity occurs when an individual defines themselves through knowledge, skills, attitudes, values, and behaviour as a part of a profession [26, 27]. Professional inclusivity e.g. students attending clinical placement, the work environment and HCP treatment and attitude towards students may affect students’ development of their professional identity [27]. It is important that students learn to be comfortable in explicitly expressing their need for help, even though it might be uncomfortable, and they may feel like a burden. Likewise, we need to teach experienced HCPs to react constructively to such calls for help and provide them with the time and framework to do so. The healthcare system and HCPs should draw more attention to students’ descriptions of experiences. This can be used to create an environment for both HCPs and students to verbalize issues and establish a safe setting for support and mentoring. Furthermore, if HCPs work consciously with their function as role-models, and if students become more aware of exchanging experiences with peers, it might help enhance a healthy development of students’ professional and emotional identity.

A third finding from our study was how students’ narrative of errors, error culture and patient complaints affected their actions and thoughts of SV. Students’ narratives contained, what we would call *myths of errors, error culture and patient complaints*.

In our study, students expected failure to be an inevitable part of their future work as a physician. However, students had a tremendous fear of making errors, causing patient injury, and being a subject of public ridicule and individual blame by colleagues and peers. Students imagined the healthcare system to be unsupportive in the case of errors and to denounce responsibility, leaving the victim with an individual responsibility. The fear of patient injury and the perception of an unsupportive healthcare system made students’ question their educational choice of profession. A Norwegian study showed students worried more than physicians about harming patients, and the majority of the students had thoughts of quitting their medical education because of the risk of harming a patient [28]. Nonetheless, students valued discussing adverse events and error-making with their network and wished a greater focus on this from HCPs and peers. Similar findings have been identified among HCPs. A Swedish study has shown that HCPs valued colleagues listening and showing empathy concerning adverse events. The HCPs stated the importance of multiple follow-ups after an adverse event, because needs could differ over time [29]. It has been suggested that HCPs experiencing personal distress because of an error are at risk of entering a vicious cycle resulting in poor patient care and elevated risk for future errors [30]. Due to this knowledge, it is essential to establish a culture, where peers and HCPs can openly talk about errors and the emotional impact.

Errors are not always caused by an individual. System-related circumstances and factors often represent a fundamental part of the underlying dynamics involved in an error. Errors are often caused by people with good intentions who accidentally make an mistake because of various factors e.g. individual, workplace, communication, technology, psychology and organisational factors [31]. An educational environment taking modern views on errors into account may contribute to a healthier development of students’ professional identity and can enhance better reaction patterns, leading to more manageable situations for students in the future. There is a need to demystify errors and other triggers for SV symptoms. Feasible initiatives could be to teach students more about the general error culture in the healthcare system, and to familiarize students with the fact that errors are typically caused and influenced by multiple factors and are not always a case of personal responsibility. Additionally, students must also learn strategies for coping with the emotional aftermath and waiting time whilst processing an error or patient complaint. Furthermore, a responsibility should be placed at an organisational level to secure offers of systematic support and follow-up for students, equal to other HCPs, despite their short and alternating clerkships. Studies describe that disclosure of errors and complaints require leadership and organisational commitment and support [32]. Furthermore, support of HCP needs to be proactive [30], which can be provided at an organisational level.

Lastly, to change the existing culture related to SV, we suggest focusing on how to change the attitude and approach towards SV in the clinical environment and to emphasize the learning perspectives and needs in the medical education.

Our findings show that students who are not met positively in uncomfortable situations tend to supress emotions and the request for help. Students’ unfortunate behaviour may extend further on to future experiences as newly graduated physicians. Especially related to medical errors, literature has shown that students with well-developed professionalism may have the personal value to help them deal with the error and potential adverse effects more honestly and efficiently [33]. We propose a more open and equal approach towards students' experiences and emotions by HCPs, colleagues, and peers early on to prevent future unhealthy behaviour and negative reactions to SV incidents and emotions. Moreover, if physicians act differently and talk more openly about the difficulties in their work-life as well as make demands related to their mental health, students’ might reflect these role-models and contribute to a healthy culture change.

We suggest implementing initiatives into the medical curriculum e.g., preparing students through greater focus on SV including hand-on tools for approaching SV experiences and emotions. Learnings activities could include lectures, case-based learning, discussion groups or simulation-based training. Similarly, the healthcare organisation must provide education on SV to HCPs for establishing a healthier work environment for themselves and students.

### Study limitations

We recruited students from the University of Copenhagen, which may have made our findings less representative of Danish students in general. Diverse composition of the patient safety education and the structure of clerkships may differ between medical programs throughout Denmark, which may affect students’ knowledge, experiences, and perceptions regarding SV phenomenon and syndrome. Recruitment of voluntary participants may produce a selection bias with participants having different characteristics from non-participants. The majority of participants were female, which are known to report more distress related to SV experiences [3]. This could lead to overestimation of the phenomenon. However, our study sample was representative for the ratio between females and males studying medicine at our institution.

At the time the interviews were conducted the interviewers (TBK, AMC) were a final year medical student and newly graduated respectively. Their equal position to the students might have given the interviewers a unique opportunity of understanding the students’ experiences, perspectives, and feelings in the interviews. The research team also included an anaesthesiologist (DØ), a psychologist (PD) and a person with a Master of arts in Philosophy (MDM) which with their professions gave nuanced perspectives to the analysis of the interviews. However, having all worked with the topic for some time and striving to improve working conditions for HCP’s, some of the authors might tend to overrate the severity of the impact of SV.

## Conclusion

Our study shows that medical students report experiencing SV similar to HCPs. Students’ emotional responses were caused by classical SV triggers, but also triggers in the educational environment; harmful interactions with individuals and feelings of self-blame. Students’ coping centred around their network, formal offers of support, and separation of personal- and work-life. Role-models were desired to legitimize and reflect students’ experiences and emotions. Students wish for more knowledge about SV phenomenon and syndrome, the SV phenomenon to be more recognised in the clinical clerkships and to establish a work environment where peers and HCPs can talk openly about adverse events, errors, and emotions. Our findings show although some triggers differ from the SV definition, these different triggers should be considered equally serious and acknowledged to ensure students’ mental well-being and the development of their professional identity for future conduct and appropriate emotional response to SV experiences as physicians. We must aim to prepare students for handling experiences and emotional responses and provide guidance to use themselves as frame of reference when experiencing being a SV. The healthcare organisation and HCPs must contribute to the ideal conditions for students to develop and stay healthy in the process.

### Supplementary Information

**Additional file 1.** Appendix 1: Interview guide.

**Additional file 2.** Appendix 2: Additional citations.

## Data Availability

The dataset used and/or analysed during the current study are available from the corresponding author on reasonable request.

## Abbreviations

HCP: Healthcare professional(s)
SV: Second victim(s)
