# Supplementary material for: Medical students’ experiences, perceptions, and management of second victim: an interview study
Source: BMC Med Educ. 2023 Oct 24;23:786. doi: 10.1186/s12909-023-04763-7 (PMC10598910; doi:10.1186/s12909-023-04763-7)
Supplement: Supplementary file 1 — Additional file 1. Appendix 1: Interview guide. [file 12909_2023_4763_MOESM1_ESM.docx]

**Appendix 1: Interview guide**

| **Topic** | **Main questions** | **Aim** |
| --- | --- | --- |
| Introduction and presentation | Presentation of interviewers.  Introduction to the concept of focus group interview.  Confirmation of participants reading introduction letter.  A summary of the term “second victim”.  Presentation of participants. |  |
| Second victim | Have you heard of the term “second victim” before today?  Have you experienced any difficult situations or second victim experiences regarding patient treatment, or do you experienced others being involved in a second victim experience?  Did you have the possibility to talk to anyone after the experience? Who? How did it happen? Did it help? | Coverage of the participants knowledge of and experiences with second victim. |
| Making errors | What considerations do you have regarding you making an error which can have serious consequences for a patient?  How do you experience the impact of your educational environment on your perception of error culture? | Coverage of the participants perception of and experience with error culture. |
| Coping with emotional distress | How do you ideally handle distress related to second victim experience or other difficult situations?  What choices, do you think, should be involved in the processing second victim related distress? | Coverage of the participants relationship to coping with emotional distress. |
| Emotions and regulation of behaviour | How do you manage your emotions in a difficult situation or a second victim experience? Is there any room for the emotions in the moment or after the moment? | Coverage of the participants involvement of emotions and the results of these consequently. |
| Aspects in a learning session | If you were on X academic year, what would you like to learn regarding second victim?  How do we assure that the topic stays relevant for clinical practice? | Coverage of the participants suggestions for potential learning needs/aspects. |
| Ending | Summary of today’s interview.  Thoughts from participants on participating in a focus group interview.  Information about re-traumatization of experiences told during the interview and contact details for help and/or questions.  Closing statements from participants and/or interviewers. |  |
|  | | |
