# Supplementary material for: Medical students’ experiences, perceptions, and management of second victim: an interview study
Source: BMC Med Educ. 2023 Oct 24;23:786. doi: 10.1186/s12909-023-04763-7 (PMC10598910; doi:10.1186/s12909-023-04763-7)
Supplement: Supplementary file 2 — Additional file 2. Appendix 2: Additional citations. [file 12909_2023_4763_MOESM2_ESM.docx]

# **Appendix 2: Additional citations**

|  | **Citation** | **Theme (subtheme)** | **Paraphrase** |
| --- | --- | --- | --- |
| 1 | *“[…] I think the reason why we describe them [adverse events] like this is because there is no room and there is no space for talking about simpler, smaller situations, like where you are misunderstood or are experiencing something less intense, but you just ignore it, because that’s what you do, and because there are so many violent shifts [one-to-one with a patient], and all consultants are so freaking arrogant and don’t have time to listen to you […]” (Interview 3)* | Contributing factors (the role of the health care professionals) | The description of adverse event and the effect of healthcare professionals on students’ way of perceiving and handling adverse events. |
| 2 | ***“****[…] I have noticed during my clinical rotations, that I want to seem competent and a bit cool, and maybe also a bit tough, look like I have the upper hand […]. I think it can be difficult, and that’s just as a student, but I think it will be even worse for me as a physician […], and sometimes admit that I want to know everything, but I can’t, and those questions, you want to ask, can be difficult to ask, because you don’t want to seem stupid.” (Interview 3)* | Contributing factors (Students’ own role) | The perception of wanting to being perfect and the fear of being vulnerable towards others. |
| 3 | *“[…] if I ask these questions or if I suddenly tell this is difficult, [and] that I think it was an uncomfortable situation, then I’ll suddenly appear less suitable for some assignments which others then will obtain […]. If I think some of these things have been difficult, then it might be easier to bring another medical student who hasn’t felt it was difficult.” (Interview 3)* | Contributing factors (Students’ own role) | Asking for help as a sign of weakness and therefore making other candidates more attractive and suitable. |
| 4 | *“[…] I was in three different hospitals and new classes all the time, so you never got to feel calm and to have some confidence on the departments […] One thing is to be thrown into a totally new hospital, a whole new department, new fellow students, and new physicians. That is on its own a really stressing situation, but when you on top of that are thrown into situations that you’re not particularly confident with, or you must acquire new competences that you don’t manage to do […] It’s not the moment, where you think ‘let me sit down and talk with this brand-new person about how difficult I think it is’.” (Interview 3)* | Contributing factors (Work environment and culture) | The feeling of lacking a base during clerkships and the influence on learning and showing emotions to brand new persons. |
| 5 | *“I also think it’s frightening, and I can sometimes have a fear of, that I can be so afraid of making an error, that when I become a physician one day and I make an error, if I dare to tell anyone, or if I dare to do something about it […].” (Interview 2)* | Contributing factors (Work environment and culture) | The fear of making errors and doubt about speaking up about it. |
| 6 | *“[…] I think that some of the thoughts I had was like ‘It’s stupid that I react this way. It shouldn’t be about me’, you feel that you are taking somebody else’s place that should’ve been given to the patients. It’s the patients who are not feeling well. It’s the patients, who are experiencing something terrible, life-threatening, traumatic. I’m just at work.” (Interview 1)* | Contributing factors (Triggers for second victim emotions) | The neglect of own emotions and needs related to patients’ suffering and experiences. |
| 7 | *“[...] I’m personally quite good at just separating the two worlds. There’s a me [name], who walks into the hospital, and there’s a [name], who like does things at the hospital [...], personally there is something, isn’t it called compartmentalise, where you can, like you can bring out a box and you can also just put it back on the shelf.” (Interview 2)* | Current coping strategies (Individual processing) | The use of “compartmentalise” where private-life and work-life I separated to minimize the amount of distress. |
|  | *“[…] if everyone knows the concept of second victim, then it would be much more relatable, instead of it being such a diffuse size with ‘are you just a sensitive person?’ or ‘do you take too much responsibility on you?’, ‘maybe I should be a little bit more tough?’. If everybody knew this problematic and everyone knew that people have experienced it one way or another, then there would be an even bigger possibility to talk it through and develop a better culture surrounding it.” (Interview 2)* | Perception on own requirements and learning needs (Expansion of knowledge) | The wish of second victim to become more well-known and relatable as a way of normalizing emotions in the work environment. |
|  | *“[...] if you could do it on each level of the hierarchy. Okay, so there’s the recent graduated, then there’s those who’ve been physicians for five years, then there’s those who’ve been for ten years but not senior physician yet, and then there’s a senior physician and a higher-higher ranked something. That would be cool.” (Interview 1)* | Perception on own requirements and learning needs (Role modelling) | The value of know HCP with different levels of experience could also be affected of second victim. |
|  | *We are practicing how to talk with patients’ multiple times, and we record it, and we practice the difficult conversation by telling actors they’re dying [...] it could also be a good starting point for talking about, how do we talk with each other about an experience [...] people are watching the same episode, but everyone has a different perception, and there should be room for that, and you should be able to talk about it in a positive way, so you can move on, and you can learn from it, but without feeling uneasy all the time.” (Interview 1)* | Perception on own requirements and learning needs (Helping others being SV) | The wish for more education on how to talk to each other about being second victim, and how to help each other. |
|  | *“[…] it wouldn’t be that hard to tell the educational supervisors on the departments […] that this is being taught [students]. You must do minimum to implement it and say this sentence [to the students], and of course be there if people need it, but that might be in the supervisor’s interest. […] like that you sense in everyday life that it’s being taken seriously, and it’s not just some academic stuff, they made up at a patient safety course.” (Interview 2)* | Wishes for future healthcare system (Impact on work culture) | The importance of second victim to become an integrated part of the work environment by educating the existing healthcare personnel. |
|  | *“[...] if you could receive more feedback like ‘okay, you were mistaken from the beginning’ or ‘it sounded like you were on the right track, but then it went wrong’, then when you graduate and start your foundational training be like ‘okay, maybe I don’t know precisely what’s wrong with the patient, but at least I know how to deal with it or tackling it’.” (Interview 1)* | Wishes for future healthcare system (Feedback) | The request for more focus on feedback on the process towards to an answer rather than the reward of finding the right answer. |
